# Baroreflex sensitivity is impaired in survivors of mild COVID‐19 at 3–6 months of clinical recovery; association with carotid artery stiffness

**DOI:** 10.14814/phy2.15845

**Published:** 2023-10-31

**Authors:** Prachi Srivastava, P. M. Nabeel, Kiran V. Raj, Manish Soneja, Dinu S. Chandran, Jayaraj Joseph, Naveet Wig, Ashok Kumar Jaryal, Dick Thijssen, Kishore Kumar Deepak

**Affiliations:** ^1^ Department of Physiology All India Institute of Medical Sciences New Delhi India; ^2^ Healthcare Technology Innovation Center Indian Institute of Technology Madras India; ^3^ Department of Electrical Engineering Indian Institute of Technology Madras India; ^4^ Department of Medicine All India Institute of Medical Sciences New Delhi India; ^5^ Department of Physiology Radboud University Medical Center Nijmegen The Netherlands

## Abstract

The association between the stiffening of barosensitive regions of central arteries and the derangements in baroreflex functions remains unexplored in COVID‐19 survivors. Fifty‐seven survivors of mild COVID‐19 (defined as presence of upper respiratory tract symptoms and/or fever without shortness of breath or hypoxia; SpO2 > 93%), with an age range of 22–66 years (27 females) participated at 3–6 months of recovering from the acute phase of RT‐PCR positive COVID‐19. Healthy volunteers whose baroreflex sensitivity (BRS) and arterial stiffness data were acquired prior to the onset of the pandemic constituted the control group. BRS was found to be significantly lower in the COVID survivor group for the systolic blood pressure‐based sequences (BRS_SBP_) [9.78 (7.16–17.74) ms/mmHg vs 16.5 (11.25–23.78) ms/mmHg; *p* = 0.0253]. The COVID survivor group showed significantly higher carotid *β* stiffness index [7.16 (5.75–8.18) vs 5.64 (4.34–6.96); (*p* = 0.0004)], and pulse wave velocity *β* (PWV_*β*_) [5.67 (4.96–6.32) m/s vs 5.12 (4.37–5.41) m/s; *p* = 0.0002]. BRS quantified by both the sequence and spectral methods showed an inverse correlation with PWV_*β*_ in the male survivors. Impairment of BRS in the male survivors of mild COVID‐19 at 3–6 months of clinical recovery shows association with carotid artery stiffness.

## 1 INTRODUCTION

COVID‐19 has been reported to produce multisystem ailments during the acute as well as post‐acute “long COVID” states (Cenko et al., 2021; Desai et al., 2022; Soriano et al., 2022). Cardiovascular consequences of COVID‐19 are attributed to a plethora of pathophysiological derangements including myocardial injury, myocarditis, endothelial dysfunction, vascular inflammation, oxidative stress, and a prothrombotic state (Behrooz et al., 2022; Nuzzi et al., 2022; Six et al., 2022). The largest cohort‐based study published to date on post‐acute cardiovascular outcomes in COVID‐19 survivors reports a substantial risk and 12‐month burden of incident cardiovascular diseases spanning ischemic, nonischemic heart diseases, and dysrhythmias emphasizing the need to integrate cardiovascular health monitoring into the care pathways of COVID‐19 survivors (Xie et al., 2022).

Various studies since the onset of the pandemic report elevation of carotid‐femoral pulse wave velocity in COVID‐19 survivors portraying an increase in regional aortic stiffness. However, there are only limited data (Szeghy et al., 2022; Zanoli et al., 2022) currently available on the COVID‐19‐associated changes in the local stiffness of central arteries. Szeghy et al. based on a study conducted in 15 survivors who were assessed 3–4 weeks after turning COVID‐19 positive reported significantly higher carotid artery stiffness in the survivors in comparison to controls. Zanoli et al. (2022) studied 90 survivors 12–48 weeks after COVID‐19 onset and reported significantly higher carotid incremental elastic modulus and lower carotid distensibility in comparison to the control group.

Examining the local stiffening of the barosensitive regions of central arteries including the carotid artery is of considerable significance in the background of studies reporting orthostatic intolerance in a significant proportion of COVID‐19 survivors (Blitshteyn & Whitelaw, 2021; Monaghan et al., 2022; Shouman et al., 2021). Local stiffening of the barosensitive regions of central arteries can render the arterial wall less deformable by the luminal pressure changes thereby reducing the mechanical gain of the baroreceptor reflex arc. The ensuing baroreflex dysfunction might explain the orthostatic intolerance, orthostatic hypotension, and postural orthostatic tachycardia syndrome reported with greater prevalence in COVID‐19 survivors. Two studies have reported baroreflex sensitivity (BRS) in COVID‐19 survivors to date. Both the studies (Skow et al., 2022; Zanoli et al., 2022) reported that BRS was comparable between the COVID‐19 survivor and control group although one of them reported significant improvement of BRS in a subset of COVID‐19 survivors followed up after 27 weeks indicating that BRS was possibly impaired in the first visit (Zanoli et al., 2022). Additionally, both studies have reported only the time domain measures of BRS and not the frequency domain estimates in low‐ and high‐frequency bands (Skow et al., 2022; Zanoli et al., 2022). The association between the stiffening of barosensitive regions of central arteries and the derangements in baroreflex function currently remains unexplored in COVID‐19 survivors. In the present study, we investigated the impact of COVID‐19 on carotid artery stiffness and its association with concomitantly measured baroreflex functions.

## 2 SUBJECTS AND METHODS

The study was conducted as a cross‐sectional observational study in the Autonomic and Vascular Function Laboratory, Department of Physiology, All India Institute of Medical Sciences, New Delhi, India. Written informed consent was obtained from all the participants prior to inclusion in the study. The study procedures were ethically approved (ref. no. IECPG‐388/23.06.2021, RT‐12/28.07.2021) by the institute's ethics committee for research on human subjects and were performed in accordance with the ethical standards as laid down in the Declaration of Helsinki (1964) and its later amendments.

COVID‐19 survivors were eligible for inclusion in the study after 1 month of clinical recovery from RT‐PCR‐positive mild COVID‐19. Mild COVID‐19 disease was defined as the presence of upper respiratory tract symptoms and/or fever without shortness of breath or hypoxia (SpO_2_ > 93%) as per AIIMS‐ICMR COVID‐19 national task force guidelines (Sharma, 2021). COVID‐19 survivors with a history of chronic cardiovascular, pulmonary, neurological, oncological, or endocrine disorders or on any chronic medications were excluded along with pregnant female participants. The rationale for restricting the recruitment to mild COVID‐19 was based on the previous reports showing greater prevalence of diabetes mellitus, hypertension, and other cardiovascular and cerebrovascular diseases in patients with moderate and severe COVID‐19 (Gao et al., 2021) which may have a direct impact on arterial stiffness and BRS. Therefore, to eliminate the confounding effect of these comorbidities and ensure the homogeneity of the COVID‐19 survivors with reference to disease severity, only survivors of mild COVID‐19 were recruited in the present study. Advertisements inviting voluntary participation of COVID‐19 survivors fulfilling the recruitment criteria were displayed as posters on and off the hospital campus and published in the local newspapers. Volunteers who contacted telephonically were assessed for their eligibility and subsequently invited to participate in the study. Eligible participants reported to a temperature‐controlled laboratory after overnight fasting and abstinence from consumption of caffeinated beverages, alcohol, and smoking for at least 12 hours and heavy exercise for 24 hours. Female participants were instructed to report in the early follicular phase based on self‐reported menstrual history. A detailed clinical history was obtained using a structured questionnaire following which the experimental procedures were performed.

The seroprevalence of COVID‐19 in India exceeded 60% after the second wave which peaked in April 2021 (Jahan et al., 2022). Based on the study conducted in the vaccine‐naïve population of rural India, George et al. reported a low case‐to‐undetected infections ratio of 1:8.65 (George et al., 2022) which necessitated the inclusion of a historical control group from the pre‐pandemic time to have a valid comparison of the outcome variables. Based on the availability of historical pre‐pandemic data for specific outcome variables, separate control groups had to be included for the comparisons of arterial stiffness and BRS. The control data for arterial stiffness parameters was obtained from a cohort of healthy volunteers who participated in a cross‐sectional community‐based study in 2016. The historical control data for BRS was obtained from a different group of apparently healthy volunteers who underwent an assessment of cardiovascular autonomic functions in our laboratory during 2016–2018. To ensure that the data acquired are technically comparable between two‐time points separated by 6–7 years, we used devices with the same hardware and software capabilities and adopted the same operating procedures for obtaining arterial stiffness and BRS data which were standardized, respectively, in 2015 (Joseph et al., 2015) and 2013 (Kaur et al., 2013). The data acquisitions were supervised by the same core team of researchers for its adherence to the standard operating procedures at both time points. However, due to logistic challenges, we could not have the same personnel acquire the data at both time points. Additionally, ARTSENS® technology, which was deployed for arterial stiffness measurements, uses an automated framework that eliminates any manual decisions or interventions by the operator thereby reducing markedly the operator‐induced variability. This was established in the detailed validation of ARTSENS® technology in 2015 (Joseph et al., 2015). It yielded measurements with greater than 85% repeatability, and the results were comparable with that of a state‐of‐the‐art automated B‐mode system—Aloka eTracking. Similarly, the process of computing BRS is automated through the software workflow and does not involve manual steps carried out by the operator. Based on the justifications stated above, we expect the data to be comparable between the two‐time points from a technical perspective.

The comparative control group (group 1) for arterial stiffness parameters consisted of 53 healthy subjects matched for age, sex, BMI, and blood pressure whose arterial stiffness data was acquired prior to the onset of COVID‐19 pandemic. In addition to a full matching on sex, an algorithm was implemented to iteratively update the error tolerance for age from 0 to ±4 years, BMI from 0 to ±2.5 kg/m^2^, and BP from 0 to ±5 mmHg, and perform matching in an automated manner. Thus, the algorithm picked up all the possible matched subjects with lower difference boundaries before ascending to the higher ones, in an iterative manner. The comparative control group (control group 2) for the comparison of BRS comprised of a separate group of 30 age‐matched male healthy volunteers. Participants of both the control groups had no history of chronic cardiovascular, pulmonary, neurological, oncological, and endocrine disorders and were not on any chronic medications at the time of assessment.

## 3 ASSESSMENT OF BAROREFLEX SENSITIVITY

Continuous noninvasive recording of ECG and blood pressure was done simultaneously for 5 min to compute BRS using spontaneous sequence and spectral methods. Participants rested for 15 min in supine position before the signals were acquired. After appropriate placement of disposable Ag‐AgCl electrodes, standard bipolar limb lead II ECG was recorded using a bipotential amplifier connected to PowerLab™ 8/35 acquisition unit and interfaced to a personal computer running the acquisition and analysis software Lab Chart Pro™ 7 (AD Instruments, Australia). ECG signal was digitally sampled at 1 kHz, and a bandpass filter (0.5–35 Hz) was applied to precondition the signal for subsequent R‐R interval extraction. Beat‐to‐beat blood pressure signal was simultaneously recorded using finger photoplethysmographic technique operating on the volume clamp principle (Finometer® MIDI, Finapres Medical Systems, Amsterdam, Netherlands). The analog output of Finometer® was fed to PowerLab™ 8/35 acquisition unit for synchronous acquisition of both the ECG and blood pressure signals. Participants were instructed to breathe spontaneously, while the recording of beat‐to‐beat blood pressure and ECG were continued for 5 min for assessment of BRS. All the recorded signals were saved for the offline analysis using Nevrokard ver.6.2.0 (Slovenia) software for the computation of BRS in both time and frequency domains.

**FIGURE 1:**
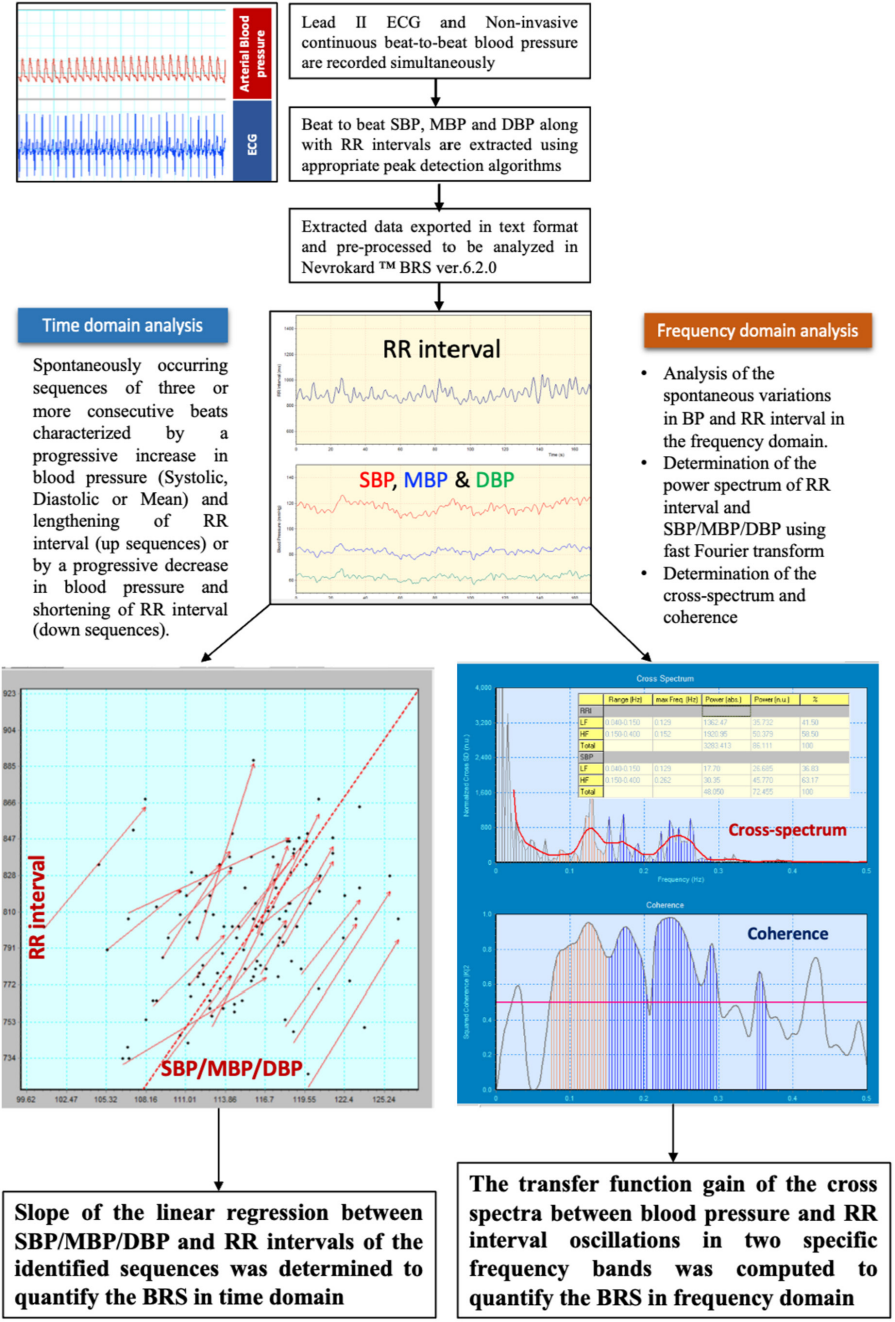
Diagram showing the steps involved in the quantification of BRS. DBP, diastolic blood pressure; MBP, mean blood pressure; SBP, systolic blood pressure.

The sequence method (time domain analysis) of BRS estimates the sensitivity of baroreceptors by analyzing the spontaneously occurring sequences of three or more consecutive beats characterized by a progressive increase in blood pressure (systolic, diastolic, or mean) and lengthening in R‐R interval (up‐sequences) or by a progressive decrease in blood pressure and shortening in R‐R interval (down sequences). The variations in R‐R intervals >5 ms, change in BP >0.5 mmHg, sequences longer than or equal to three beats, and a correlation coefficient of sequences >0.85 were the criteria used for the inclusion of sequences. The slope of the regression line between the R‐R interval and blood pressure values of the identified sequences signifies the baroreflex sensitivity in the time domain (La Rovere et al., 2008; Parati et al., 2000). The identification of sequences and further analysis and computation of BRS in the time domain were automatically performed by the software package Nevrokard™ BRS ver.6.2.0 (Slovenia). The spectral method computes the sensitivity of baroreceptor reflex based on the analysis of spontaneous variations in BP and R‐R interval in the frequency domain (La Rovere et al., 2008; Parati et al., 2000). The transfer function gain of the cross spectra between blood pressure and R‐R interval oscillations in two specific frequency bands was computed to quantify the alpha coefficients of BRS at low‐frequency (alpha‐LF) band (0.04–0.15 Hz) and high‐frequency (alpha‐HF) band (0.15–0.4 Hz). Figure 1 diagrammatically depicts the steps involved in the quantification of BRS.

## 4 ASSESSMENT OF LOCAL CAROTID ARTERY STIFFNESS

Stiffness of the common carotid artery was assessed using the ARTSENS® plus device (Healthcare Technology Innovation Center, IIT Madras, India)—a clinically validated, image‐free, ultrasound‐based arterial wall tracking technology (Joseph et al., 2020). The subjects were allowed to rest for 10 min in supine posture before the measurement was carried out. The resting brachial blood pressure of the subject was measured using an automated oscillometric BP measurement module integrated with the ARTSENS® plus device. The best palpable point of carotid artery pulsation was then identified using fingers. After applying the ultrasound gel, the tip of the image‐free ultrasound probe (10 Hz) was placed superficially over the best palpable point of carotid pulsation. The graphical user interface of the device displayed the A‐mode ultrasound echo frames, the quality metrics of wall echoes, and the recorded carotid distension waveforms, online. The probe orientation was adjusted to get strong and sharp distinct echoes from both the near and far walls of the artery as visually guided by the device and displayed on the computer screen. An on‐screen progress bar fills up while capturing high‐fidelity distension waveform, and the device automatically generates the results upon acquisition of the desired‐quality cycles for a target number of beats. Carotid artery stiffness indices were derived by the ARTSENS® software using clinically accepted formulae (Mackenzie et al., 2002) based on the changes in the carotid artery diameter over the cardiac cycle, D_S_ at peak‐systole and D_D_ at end‐diastole, and the corresponding carotid pressures, P_S_ and P_D_, which were nonlinearly scaled (Meinders & Hoeks, 2004; Vermeersch et al., 2008) from Oscillometric brachial BP. These include:

*Stiffness index* (*β*): The parameter is derived directly from the exponential pressure–diameter relationship of an artery, and is a dimensionless index:

(1) β=lnPSPDDS−DDDD.

*Pressure–strain elastic modulus* (*E*_*p*_): A metric defining the ratio of stress to strain, in terms of easily measurable pressure and diameter parameters, as (expressed in units of kPa):

(2) $$E_{p}=\frac{P_{S}-P_{D}}{\left(\frac{D_{S}-D_{D}}{D_{D}}\right)}.$$

*Arterial compliance* (*AC*): The metric represents an inverse of elastic modulus, given as the ratio of absolute change in cross‐sectional area within a cardiac cycle to the change in pressure, expressed in units of mm^2^kPa^−1^.

(3) $$\mathrm{AC}=\frac{\frac{\pi }{4}\;\left(D_{S}^{2}-D_{D}^{2}\right)}{\left(P_{S}-P_{D}\right)}.$$

*One‐point pulse wave velocity* (PWV_*β*_): The speed of blood pulse propagation through a point local to an arterial segment. The measure can be theoretically evaluated from the stiffness index, as

(4) $${\mathrm{PWV}}_{\beta }=\sqrt{\frac{P_{D}\;\beta }{2\;\rho }},$$

where the blood mass density *ρ* ≈ 1050 kgm^−3^ and is expressed in terms of m s^−1^.

### 4.1 Statistical analysis

No prior calculation of sample size was carried out for the present study as the limiting factor was the availability of adequate number of age, sex, BMI, and blood pressure‐matched control subjects whose measurements have been carried out before January 2020. Each parameter was tested for the normality of distribution using D'Agostino‐Pearson normality test. Based on the result of the normality test, data of normally distributed variables were represented as mean ± standard deviation and, for those of the variables that did not fall in a Gaussian distribution, median (25th centile–75th centile) was used as the standard norm for representing the summary statistics across the manuscript. Unpaired *t* test or Mann–Whitney test was used as applicable to compare the outcome variables between the control and the COVID‐19 survivor groups. In the COVID‐19 survivor group, the time domain and frequency domain parameters of BRS were assessed for their correlation with the carotid artery stiffness measures using Pearson or Spearman tests as applicable depending on the distribution of the variables included. A *p* value of less than 0.05 was considered statistically significant. All statistical analysis was done by using GraphPad Prism version 9.4.1 for Windows (GraphPad Software, Inc.).

## 5 RESULTS

Fifty‐seven COVID‐19 survivors who were diagnosed with RT‐PCR‐positive mild COVID‐19 participated in the study at least 1 month after clinical recovery from the acute phase of the illness. All the participants were managed in home isolation during the acute phase of the illness and self‐reported to maintain O_2_ saturation ≥94%. None of the patients suffered from any complications due to COVID‐19 which required hospitalization or outpatient care. The demographic and relevant clinical characteristics of the COVID survivor group and the control group 1 are shown in Table 1.

**TABLE 1:** Demographic and clinical characteristics of the COVID survivor group and matched historical healthy control group (group 1 for comparison of arterial stiffness parameters).

Characteristics	Healthy control group 1 (*n* = 53)	COVID survivor (*n* = 57)	*p* Value
Age (years)	33 (30–39.5)	34 (30–40.5)	0.70^#^
Sex (M:F)	29:24	32:25	>0.99^θ^
Weight (kg)	65.9 (58.3–75.9)	64.0 (57.5–78.0)	0.96^#^
Height (cm)	163.0 (153.5–170.0)	162.0 (153.5–172.5)	0.58^#^
BMI (kg/m^2^)	25.4 (23.6–26.4)	24.7 (23.0–26.7)	0.45^#^
Systolic blood pressure (mmHg)	114.6 ± 10.7	115.9 ± 12.8	0.55^ε^
Diastolic blood pressure (mmHg)	72.3 ± 7.5	73.4 ± 8.5	0.45^ε^
Pulse pressure (mmHg)	42.3 ± 8.2	42.5 ± 7.4	0.84^ε^
Heart rate (beats per minute)		75 (67–84)	
Time point of assessment (weeks after diagnosed for COVID‐19)	–	20 (10–28)	
Symptoms at the time of assessment (no. of patients showing each symptom; % in parentheses)	–		
Cough		6 (11)	
Weakness		14 (25)	
Breathlessness while walking		3 (5)	
Breathlessness at rest		1 (2)	
Anxiety		4 (7)	
Difficulty to concentrate		4 (7)	
Palpitation		3 (5)	
Difficulty in performing activities of daily living		3 (5)	

*Note*: ^#^Mann–Whitney test, ^θ^Fisher's exact test, ^ε^Unpaired *t* test.

**FIGURE 2:**
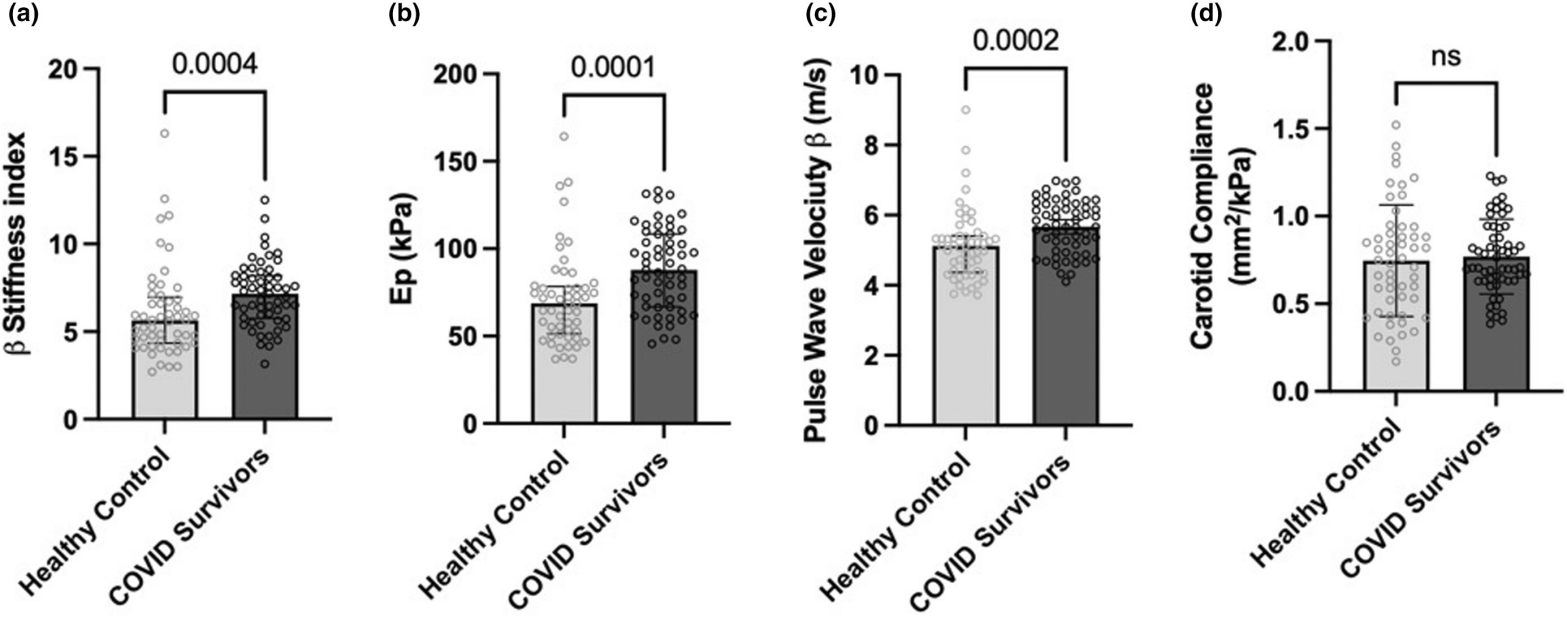
Comparison of arterial stiffness markers between the COVID survivor and matched healthy control group. Individual bar diagrams represent (a) *β* stiffness index, (b) pressure–strain elastic modulus (Ep), (c) pulse wave velocity‐*β*, and (d) area compliance of the carotid artery. Error bars represent 25th centile–75th centile in (a–c) and standard deviation in (d). Mann–Whitney test is used for the statistical comparisons between the groups in (a–c); Unpaired *t* test is used in (d).

The COVID survivor group showed significantly higher carotid *β* stiffness index [median (25th percentile–75th percentile) of 7.16 (5.75–8.18) vs 5.64 (4.34–6.96) for the healthy control group; (*p* = 0.0004)], carotid pressure–strain elastic modulus [87.97 (66.68–108.30) kPa vs 68.79 (51.46–78.42) kPa; *p* = 0.0001], and pulse wave velocity *β* [5.67 (4.96–6.32) m/s vs 5.12 (4.37–5.41) m/s; *p* = 0.0002]. Carotid arterial compliance was not different between the two groups [0.71 (0.63–0.94) mm^2^/kPa vs 0.75 (0.49–0.92) mm^2^/kPa; *p* = 0.55]. The comparison of arterial stiffness markers is shown in Figure 2.

**FIGURE 3:**
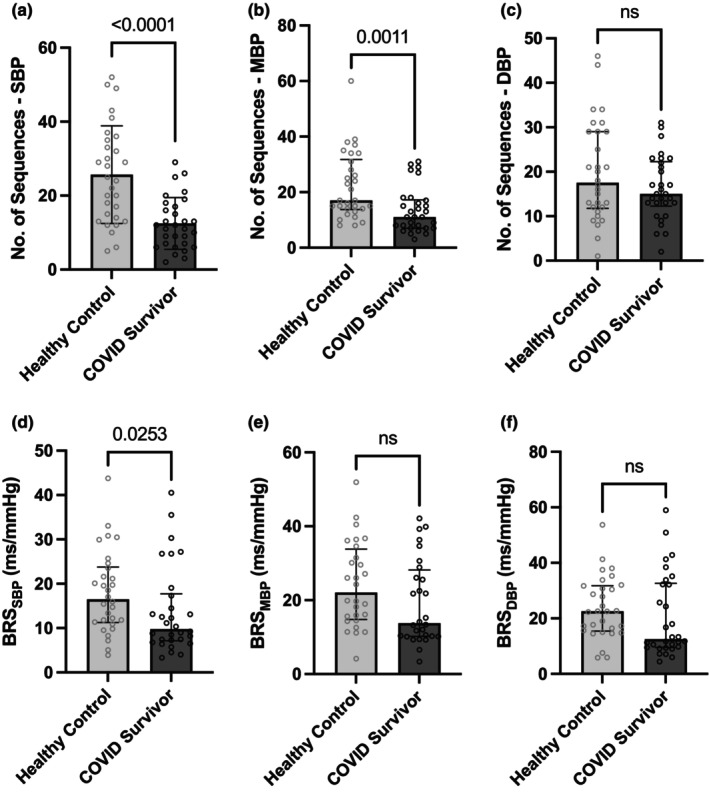
Comparison of the time domain measures of BRS computed by the spontaneous sequence between the COVID survivor and matched healthy control group. (a–c) represents the number of sequences and (d–f) the BRS quantified, respectively, for SBP‐, MBP‐, and DBP‐based analysis. (BRS, baroreflex sensitivity; DBP, diastolic blood pressure; MBP, mean blood pressure; SBP, systolic blood pressure). Error bars represent standard deviation in (a) and 25th centile–75th centile in (b–f). Unpaired *t* test is used for the statistical comparison between the two groups in (a); Mann–Whitney test is used in (b–f).

Due to the paucity of pre‐pandemic historical control data of healthy volunteers, successful age‐matching could only be done for 30 male COVID survivors for the analysis of BRS data. The demographic and physiological characteristics of this subset of COVID survivors and the matched healthy control group (group 2; not drawn from control group 1) are depicted in Table 2. Figure 3 depicts the comparative analysis of BRS data between the subset of COVID survivors and the age‐matched healthy control group. Time domain measures of BRS obtained by sequence method showed significantly lower numbers of baroreflex sequences in the COVID survivor group in comparison to the control group for both systolic (12 ± 7 vs 26 ± 13; *p* < 0.0001) and mean blood pressure [11 (7–17) vs 17 (14–32); *p* = 0.0011] based analysis (Figure 3). Further, the BRS quantified was found to be significantly lower in the COVID survivor group for the systolic blood pressure‐based sequences (BRS_SBP_) [9.78 (7.16–17.74) ms/mmHg vs 16.5 (11.25–23.78) ms/mmHg; *p* = 0.0253] but comparable for both mean blood pressure and diastolic blood pressure‐based sequences with reference to the age‐matched control group (Figure 3). We further investigated the contribution of up‐sequences versus down sequences toward the observed changes in the number of sequences and BRS_SBP._ The numbers of both up and down sequences were significantly lower in the COVID survivor group (6 ± 3 vs 13 ± 6; *p* < 0.0001 for up‐sequences and 7 ± 5 vs 12 ± 8; *p* = 0.0037 for the down sequences) in comparison to the control group (unpaired *t* test). BRS_SBP_ for down sequences was found to be significantly lower in the COVID survivor group [9.25 (6.75–13.40) ms/mmHg vs 16.48 (10.35–21.93) ms/mmHg; *p* = 0.0073; Mann–Whitney test], while BRS_SBP_ for up‐sequences did not differ significantly between the two groups [10.14 (5.56–19.31) ms/mmHg vs 16.38 (9.17–25.61) ms/mmHg; *p* = 0.1681; Mann–Whitney test]. The frequency domain measures of BRS were found to be not significantly different between the two groups (Table 3). However, both alpha HF_SBP_ (*r* = 0.785; *p* < 0.001) and alpha LF_SBP_ (*r* = 0.652; *p* < 0.001), the spectral measures of BRS, correlated strongly with the BRS_SBP_ quantified by the sequence method (Spearman correlation analysis).

**TABLE 2:** Demographic and physiological characteristics of the COVID survivor group and the matched historical healthy control group (group 2 for comparison of baroreflex sensitivity).

Characteristics	Healthy control group 2 (*n* = 30)	COVID survivor (*n* = 30)	*p* Value
Age (years)	33 ± 6	33 ± 6	>0.99^ε^
Weight (kg)	74.6 ± 8.2	73.1 ± 8.8	0.51^ε^
Height (cm)	169.0 (167.0–172.0)	171.0 (167.0–176.5)	0.19^#^
BMI (kg/m^2^)	24.8 (23.6–28.1)	25.1 (23.4–26.9)	0.36^#^
Systolic blood pressure (mmHg)	122.7 ± 10.8	119.2 ± 10.3	0.23^ε^
Diastolic blood pressure (mmHg)	69.1 ± 9.1	74.5 ± 7.2	**0.02** ^ε^
Heart rate (beats per minute)	80 (74–84)	75 (66–83)	0.06^#^

*Note*: ^ε^Unpaired *t* test, ^#^Mann–Whitney test. *p* value in bold indicates statistical significance

**TABLE 3:** Comparison of the frequency domain (spectral) measures of BRS between the COVID survivor and control group.

Parameter	Healthy control (group 2; *n* = 30)	COVID survivor (*n* = 30)	*p* Value
Alpha LF_SBP_ (ms/mmHg)	3.03 ± 1.51	3.56 ± 2.57	0.35^ε^
Alpha HF_SBP_ (ms/mmHg)	4.65 ± 2.89	4.31 ± 3.46	0.69^ε^
Alpha LF_MBP_ (ms/mmHg)	3.49 (2.65–4.46)	3.90 (2.30–6.74)	0.43^#^
Alpha HF_MBP_ (ms/mmHg)	6.85 (3.80–10.43)	5.45 (3.33–10.82)	0.41^#^

*Note*: ^#^Mann–Whitney test, ^ε^Unpaired *t* test.

**FIGURE 4:**
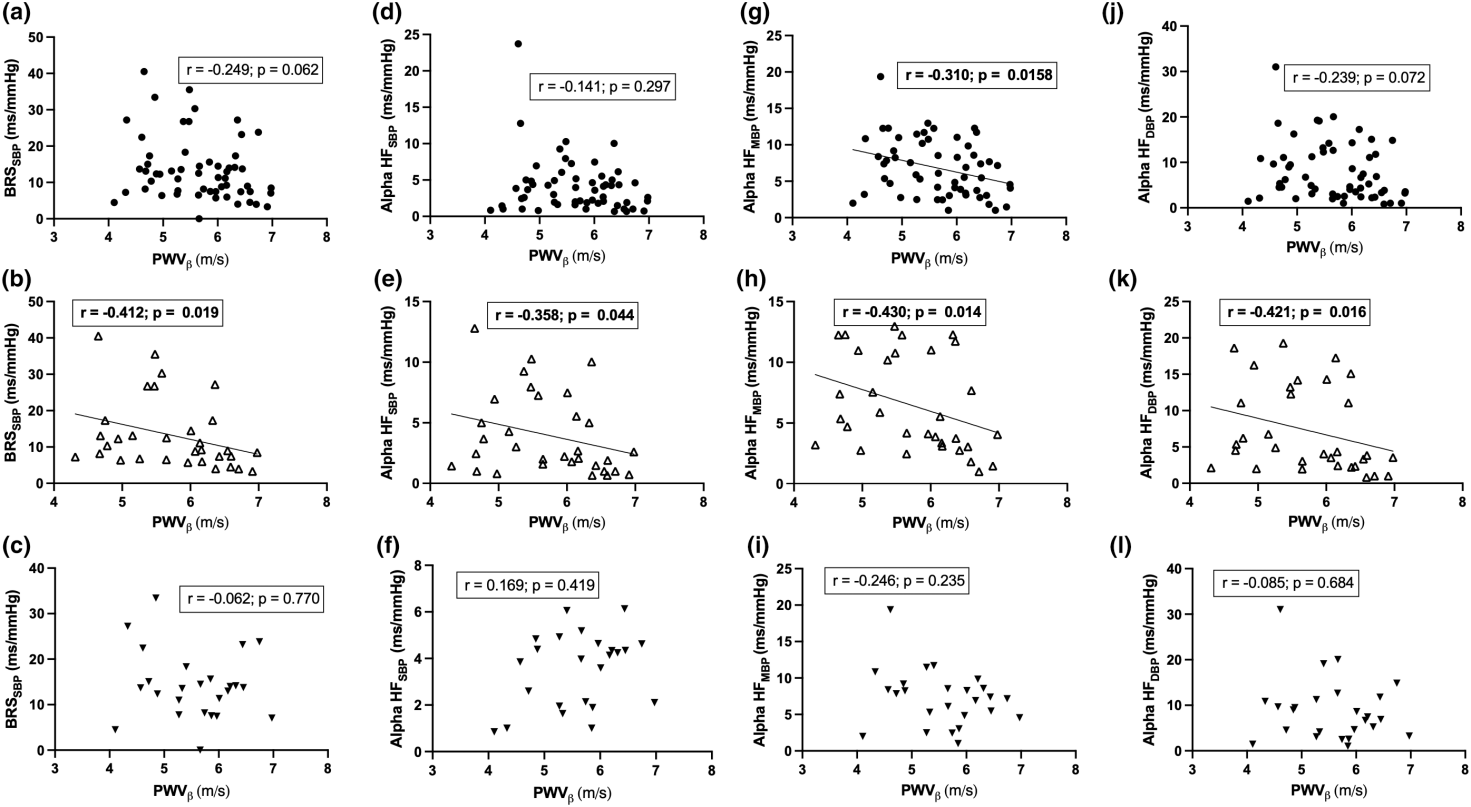
Correlations between carotid PWV‐*β* and the time and frequency domain parameters of BRS in the COVID‐19 survivor group. Individual scatter plots represent BRS_SBP_ (a–c), alpha HF_SBP_ (d–f), alpha HF_MBP_ (g–i), and alpha HF_DBP_ (j–l) in the COVID‐19 survivor group (a, d, g, j), in the male subset of COVID‐19 survivor group (b, e, h, k), and in the female subset of COVID‐19 survivor group (c, f, i, l). Boxes overlaid on each of the plots show the respective Spearman correlation coefficients and the *p* values.

In univariate correlation analysis between carotid stiffness measures and BRS for the COVID‐19 survivor group, carotid PWV‐beta and pressure–strain elastic modulus (Ep) were found to be negatively correlated with the frequency domain measure, BRS ‐ alpha HF_MBP_ (*r* = −0.31, *p* = 0.0158 and *r* = −0.289, *p* = 0.029, respectively; Spearman correlation analysis, Figure 4 & Table 4). The results of the correlation analysis between carotid artery stiffness measures and the entire set of time domain and frequency domain parameters of BRS are represented in Table 4. Since the comparison of BRS measures with matched healthy controls could only be done for the male subset of COVID‐19 survivors wherein we found statistically significant differences, we did separate correlation analyses for the male and female subsets of COVID‐19 survivors (Table 4 and Figure 4). Interestingly, in the male subset of COVID‐19 survivors, we found significant negative correlations between PWV_*β*_ and BRS_SBP_ (*r* = −0.412; *p* = 0.019), BRS_MBP_ (*r* = −0.385; *p* = 0.029), BRS_DBP_ (*r* = −0.371; *p* = 0.036), Alpha HF_SBP_ (*r* = −0.358; *p* = 0.044), alpha HF_MBP_ (*r* = −0.430; *p* = 0.014), and alpha HF_DBP_ (*r* = −0.421; *p* = 0.016); Spearman correlation analysis, Table 4 and Figure 4. Carotid artery pressure–strain elastic modulus also showed a significant negative correlation with alpha HF_MBP_ (*r* = −0.363; *p* = 0.041) and alpha HF_DBP_ (*r* = −0.354; *p* = 0.047) in the male subset of COVID‐19 survivors (Table 4). However, none of the measures of carotid artery stiffness showed any statistically significant correlations with the time domain or frequency domain parameters of BRS in the female subset of COVID‐19 survivors (Spearman correlation analysis, Table 4 and Figure 4). Figure 4 shows the graphical plots of relevant correlations of PWV_*β*_ with BRS parameters in the COVID‐19 survivor group, the male, and the female subsets of COVID‐19 survivors. Taking cognizance of the discrepancy in the results of correlation analysis in the male versus female subset of COVID‐19 survivors, we compared the measures of carotid stiffness and BRS between these two subsets and found none of these parameters to be statistically different from each other (data not shown). However, it was noted that the median time period after which the vascular and baroreflex functions were assessed post‐recovery from the acute phase of COVID‐19 stood longer in the female subset in comparison to the male subset of COVID‐19 survivors [5 (2.5–7) vs 2 (2–5) months; *p* = 0.0328; Mann–Whitney test].

**TABLE 4:** Univariate correlations between measures of carotid stiffness and BRS in the COVID‐19 survivor group.

Carotid stiffness measures	BRS_SBP_	BRS_MBP_	BRS_DBP_	Alpha LF_SBP_	Alpha HF_SBP_	Alpha LF_MBP_	Alpha HF_MBP_	Alpha LF_DBP_	Alpha HF_DBP_
Correlations in the COVID‐19 survivor group (males and females combined), *n* = 57									
*β* stiffness index	−0.126	−0.123	−0.101	−0.054	−0.028	−0.161	−0.205	−0.170	−0.113
Pressure–strain elastic modulus	−0.241	−0.203	−0.214	−0.097	−0.116	−0.202	**−0.289** (*p* = 0.029)	−0.223	−0.210
Compliance	0.036	−0.112	−0.118	0.114	−0.050	0.094	−0.031	0.092	−0.136
Pulse wave velocity‐*β*	−0.249	−0.212	−0.245	−0.105	−0.141	−0.209	**−0.310** (*p* = 0.0158)	−0.233	−0.239
Correlations in the male subset of COVID‐19 survivors; *n* = 32									
*β* stiffness index	−0.215	−0.185	−0.155	−0.035	−0.160	−0.052	−0.188	−0.101	−0.214
Pressure–strain elastic modulus	−0.346	−0.324	−0.304	−0.175	−0.288	−0.213	**−0.363** (*p* = 0.041)	−0.259	**−0.354** (*p* = 0.047)
Compliance	0.034	−0.028	0.098	0.058	0.059	0.100	0.081	0.068	0.079
Pulse wave velocity‐*β*	**−0.412** (*p* = 0.019)	**−0.385** (*p* = 0.029)	**−0.371** (*p* = 0.036)	−0.228	**−0.358** (*p* = 0.044)	−0.274	**−0.430** (*p* = 0.014)	−0.315	**−0.421** (*p* = 0.016)
Correlations in the female subset of COVID‐19 survivors; *n* = 25									
*β* stiffness index	−0.059	−0.083	−0.063	−0.057	0.157	−0.284	−0.333	−0.253	−0.032
Pressure–strain elastic modulus	−0.090	−0.033	−0.148	0.014	0.165	−0.227	−0.243	−0.229	−0.069
Compliance	0.169	−0.242	−0.252	0.032	−0.202	0.065	−0.168	0.053	−0.255
Pulse wave velocity‐*β*	−0.062	0.001	−0.178	0.062	0.169	−0.165	−0.246	−0.165	−0.085

*Note*: Spearman correlation coefficients are shown in the table. Bolded coefficients indicate the statistically significant correlations along with the *p* values in parentheses.

Age, sex, BMI, presence or absence of “long COVID” symptoms, and the interval between the onset of COVID‐19 and the time point of laboratory assessment of arterial stiffness and BRS did not show any association with the parameters of carotid stiffness or BRS.

## 6 DISCUSSION

The present study investigated the impact of COVID‐19 on carotid artery stiffness and its association with concomitantly measured baroreflex functions.

Our COVID‐19 survivor group consisted of 57 participants who survived mild COVID‐19 and were managed in home isolation during the acute symptomatic phase of the infection. None of the patients were hospitalized for any COVID‐19‐related sequelae following their recovery from the acute phase of the infection. Twenty survivors (35%) reported the presence of one or more of the “long COVID symptoms” at the time of vascular assessment with weakness being the most frequently reported symptom (25%). We observed significantly higher carotid *β* stiffness index, carotid pressure–strain elastic modulus, and pulse wave velocity‐*β* in COVID‐19 survivors in comparison to age, sex, BMI, and BP‐matched healthy controls (Figure 2). This signifies a greater carotid artery stiffness in the COVID‐19 survivor group. Our observations are consistent with earlier reports by Szeghy et al. (2022) and Zanoli et al. (2022). It is noteworthy that higher stiffness was observed in survivors of mild COVID‐19 who were managed in home isolation and were free of any comorbidities. Similar was the clinical profile of COVID‐19 survivors in the study done by Szeghy et al. (2022) who also reported greater carotid artery stiffness in nonhospitalized survivors of mild COVID‐19. Interestingly, carotid area compliance was not significantly different between the COVID‐19 survivor and control groups in the present study (Figure 2). This could probably be linked to the fact that, unlike carotid *β* stiffness index and carotid pressure–strain elastic modulus, carotid area compliance does not normalize the two‐dimensional changes in the area to the original diastolic area (Equations (1), (2), (3) above in subjects and methods section). Normalization of the change in dimension to the baseline dimension qualifies *β* stiffness index and pressure–strain elastic modulus as better markers of arterial elastic property in comparison to compliance as they are derived based on the stress–strain relationships. This theoretical premise is also supported by experimental evidence where in carotid artery compliance and *β* stiffness index have been shown to behave differentially to a systemic intervention in human subjects (Sugawara et al., 2014). The finding that pulse pressure (a surrogate marker of arterial stiffness if measured centrally) is not significantly different between the two groups (Table 1) may be attributed to the fact that controls in the current study were matched for both systolic and diastolic blood pressure with a narrow margin of tolerance of 5 mmHg with the COVID‐19 survivor group. Given that the majority of carotid artery stiffness measures were significantly higher in the COVID‐19 survivor group, the equivalence of pulse pressure may seem contradictory. However, it is important to highlight that due to the pressure amplification brought on by the wave propagation from the center to the periphery, peripheral pulse pressure (measured and reported in the present study) does not accurately reflect the central pulse pressure, particularly in young and middle‐aged individuals (Mackenzie et al., 2002) (median age of the combined pool of participants is 33.5 years in the present study). Additionally, the area compliance of the carotid artery—the parameter that is proximately linked to central pulse pressure in an inverse manner—was found to be not significantly different between the COVID survivor and control groups. Due to technical constraints, we were unable to record central pulse pressure in the current investigation, and as a result, we do not have the data necessary to discuss further on this aspect.

The BRS measured by sequence method using SBP sequences was found to be significantly lower in the male COVID survivors in comparison to age‐matched healthy male subjects (Figure 3). However, on further analysis, it was observed that this outcome was majorly influenced by BRS for the down sequences which remained significantly lower in the male COVID‐19 survivor group in comparison to matched controls, whereas BRS for up‐sequences was not statistically different between the two groups. One of the plausible mechanisms behind this preferential involvement of BRS for down sequences deserves discussion in association with two other salient observations (Table 2) 1. Higher diastolic BP indicating greater total peripheral resistance (TPR) in the male subset of COVID‐19 survivors in comparison to matched healthy controls; 2. a trend of resting heart rate being lower in the male COVID‐19 survivors in comparison to matched controls possibly implying greater vagal tone. The impact of greater TPR on enhancing MBP has been balanced and possibly nullified by the greater vagal tone which tends to reduce heart rate and cardiac output. As a consequence, the prevailing greater vagal tone would inhibit the R‐R interval responses to hypotensive episodes culminating in a lower BRS being estimated for the down sequences.

In addition to the observed differences in the BRS estimates, interestingly, the number of sequences comprising simultaneously recorded BP and R‐R interval signals that showed baroreflex engagement as per the pre‐defined criteria was also markedly lower in the male COVID‐19 survivors for both SBP‐ and MBP‐based sequences (Figure 3). Consistent with previous experimental studies (Bertinieri et al., 1988), a lower number of baroreflex‐coupled sequences in COVID‐19 survivors could indicate a lesser probability of engagement of arterial baroreflex in comparison to matched controls thereby indicating baroreflex dysfunction. However, spectral indices of BRS were found to be not significantly different between the COVID‐19 survivors and the control group (Table 3). We find largely statistical reasons attributed to greater data variance in the spectral measures as the reason for missing any statistically significant differences for these parameters between the COVID‐19 survivor group and the control group due to the low sample size in the present study. This is corroborated by the strong positive correlation we observed between the BRS computed by spectral versus sequence method in the COVID‐19 survivor group indicating that the data variances across subjects were similar and followed the same trend. However, the coefficient of variations for alpha HF_SBP_ and alpha LF_SBP_ were considerably high, respectively, at 80% and 72% (Table 3). Retrospectively calculated statistical power for the given sample size to detect any difference in the spectral measures of BRS with that of the control group as statistically significant was as low as 6.9% and 15.9%, respectively, for alpha HF_SBP_ and alpha LF_SBP_.

There have been a couple of previous studies that reported BRS measured in COVID‐19 survivors. Contradicting the findings of the present study, Skow et al. (2022) reported that BRS measured by sequence method was comparable between 23 COVID‐19 survivors (Omicron variant) and 13 healthy volunteers who never had COVID‐19 (based on self‐report). Zanoli et al. (2022) by using a modified version of the sequence method (cross‐correlation method) reported that all COVID‐19 survivors (*n* = 90) had BRS at their first visit statistically comparable with the control group. However, a randomly chosen subset of COVID‐19 survivors who were reassessed 27 weeks after the first visit showed significant improvement in BRS. The improvement might signify the course of recovery from an already impaired BRS. While Zanoli et al. studied COVID‐19 survivors who were hospitalized with moderate/severe COVID‐19 at least 12 weeks after the onset of symptoms, Skow et al. included nonhospitalized participants who were diagnosed with mild/moderate COVID‐19 and were assessed on an average at 4 weeks after the diagnosis. Although the clinical profile of COVID‐19 survivors in our study is similar to that of Skow et al., with all participants being survivors of mild COVID‐19, the time point of BRS assessment since diagnosis (median = 20 weeks) in our study matches with that of Zanoli et al. It is noteworthy that there are some salient differences that can challenge the validity of direct comparisons of the findings of the present study with those of Skow et al. and Zanoli et al. The COVID‐19 survivor group and the comparative control group of both these studies comprised of both males and females (females forming the majority in the study by Skow et al.). However, in the present study, BRS measures of the male subset of COVID‐19 survivors are compared with that of matched healthy male controls which marks the findings reported to be specific to the males. Moreover, the study by Zanoli et al. included hospitalized patients who presumably experienced severe effects of COVID‐19 than that of ours.

The central mechanistic theme of the present study was to investigate the relationship between arterial stiffness and BRS in COVID‐19 survivors which was linked to the working hypothesis that stiffening of the barosensitive segments of the arterial tree would be associated with impaired BRS. The inverse correlations we observed between one of the spectral indices of BRS (alpha HF_MBP_) with carotid PWV‐*β* and carotid pressure–strain elastic modulus are in support of this hypothesis albeit the fact that a similar relationship could not be observed with the BRS obtained by sequence method in the entire pool of COVID‐19 survivors (Table 4 and Figure 4). Since the comparison of BRS measures with matched healthy controls could only be done for the male subset of COVID‐19 survivors wherein we found statistically significant differences, we did separate correlation analyses for the male and female subsets of COVID‐19 survivors (Table 4 and Figure 4). This revealed interesting observations which included consistent and stronger negative correlations of PWV_*β*_ with multiple time and frequency domain measures of BRS in the male subset of COVID‐19 survivors, while the female subset showed no such correlations. It is noteworthy that in the male subset of COVID‐19 survivors, only the alpha coefficient of the high‐frequency band (0.15–0.4 Hz) displayed correlations with PWV_*β*_ among the spectral parameters of BRS. A recent study by Silva et al. (2019) reports that sequence method primarily determines the buffering responses in pulse interval/R‐R interval induced by the high‐frequency (HF) oscillations in the blood pressure—consistent with our finding of PWV_*β*_ showing correlations with BRS estimated by sequence method and the high‐frequency (HF) alpha coefficient computed by the spectral method. The sex‐dependent differences in the correlations intrigued us to extend our investigation into evaluating any differences in the arterial stiffness and BRS measures between the male and the female subsets of COVID‐19 survivors. However, none of the comparisons spanning the entire set of arterial stiffness and BRS measures emerged as statistically significant. Although this finding could be attributed to sub‐optimal sample size (especially with reference to the female subset; *n* = 25) for adequately powered statistical comparisons between the male and female subsets for the study parameters, there was a notable difference between the two subsets characteristically in relation to the time period after which vascular and BRS measurements were carried out post‐recovery from the acute phase of COVID‐19. Female participants on average were assessed at a considerably later time point than the male participants probably leading to a greater recovery and subduing of the associations between arterial stiffening and baroreflex functions in the female participants. The only available report on the association between BRS and arterial stiffness in COVID‐19 survivors is from the study by Zanoli et al. who reported inverse correlation between aortic PWV and BRS consistent to what we observed in the present study. However, the study does not comment on any sex‐dependent differences in the correlations observed as reported in the present study.

The findings of the present study have wider clinical implications with reference to the possible pathophysiological basis of orthostatic manifestations in COVID‐19 survivors. Ever since “long COVID” has been recognized as a clinical entity, COVID‐19 survivors presenting with orthostatic intolerance have been increasingly diagnosed with orthostatic hypotension and postural orthostatic tachycardia syndrome when subjected to laboratory‐based hemodynamic evaluation (Blitshteyn & Whitelaw, 2021; Monaghan et al., 2022; Shouman et al., 2021). The preferential/isolated deficit in BRS for down sequences (indicating a blunted baroreflex response to hypotensive episodes) as reported in the present study might explain the physiological basis of the occurrence of postural hypotension in patients recovering from long “COVID.”

The major strength of the present study is the comprehensive analysis and reporting of both the time domain and frequency domain indices of BRS in COVID‐19 survivors which none of the studies have done so far. Although earlier studies have reported BRS quantified by the sequence method, ours is the first investigation to explore further into any potential differences in the BRS determined for “up‐sequences” versus “down sequences” which has major physiological implications in the wake of orthostatic intolerance reported in COVID‐19 survivors. Our COVID‐19 survivor group is homogenous, especially with reference to the severity of COVID‐19 and the absence of any comorbidities that could independently affect arterial stiffness or BRS. This adds strength to attribute the observed alterations in the study parameters to COVID‐19. Like most of the previous reports on COVID‐19 survivors, we also used pre‐COVID historical control data to ensure the validity of the comparative analysis. Additionally, for the carotid stiffness comparisons, we used an automated iterative algorithm to choose a control group from a preexisting database matched for age, sex, BMI, and blood pressure. Blood pressure matching has not been done by any of the previous studies although they have statistically accounted for its influence in data analysis (Zanoli et al., 2022).

The present study suffers multiple limitations including, wide variations in the time point of assessment after COVID‐19 diagnosis owing to feasibility constraints, comparative analysis of the BRS data being performed only for the male subset which limits its generalizability and unavailability of any biochemical markers of inflammation or disease severity during the acute phase of COVID‐19 and markers of background cardiovascular risk including lipid profile as the patients were managed in home isolation and did not undergo any blood investigations. Additionally, since genomic sequencing to identify the COVID‐19 variant causing the infection was not routinely performed in our setting, we did not have the data on the same to draw any such associations with the findings reported.

## 7 CONCLUSION

To conclude, surviving mild COVID‐19 is associated with higher carotid artery stiffness and impaired arterial baroreflex sensitivity in the absence of any comorbidities. The inverse correlation observed between carotid stiffness and BRS—a finding more prominently and consistently observed in the male COVID‐19 survivors and not in females—might signify the association of post‐COVID stiffening of barosensitive regions of central arteries in a sex‐dependent manner with baroreflex dysfunction.

## ETHICS STATEMENT

The study procedures were ethically approved (ref. no. IECPG‐388/23.06.2021, RT‐12/28.07.2021) by the institute’s ethics committee for research on human subjects, All India Institute of Medical Sciences, New Delhi and were performed in accordance with the ethical standards as laid down in the Declaration of Helsinki (1964) and its later amendments.
